# Lewis Y regulates signaling molecules of the transforming growth factor β pathway in ovarian carcinoma-derived RMG-I cells

**DOI:** 10.3892/ijo.2011.1296

**Published:** 2011-12-13

**Authors:** FEI-FEI LI, JUAN-JUAN LIU, DA-WO LIU, BEI LIN, YING-YING HAO, JIAN-PING CONG, LIAN-CHENG ZHU, SONG GAO, SHU-LAN ZHANG, MASAO IWAMORI

**Affiliations:** 1 Department of Obstetrics and Gynecology, Shengjing Hospital of China Medical University, Shenyang 110004; 2 Department of Obstetrics and Gynecology, Shandong Provincial Hospital Affiliated to Shandong University, Jinan 250021, P.R. China; 3 Department of Biochemistry, Faculty of Science and Technology, Kinki University, Osaka 577-8502, Japan

**Keywords:** Lewis Y, p42/44 mitogen-activated protein kinase, phosphoinositide 3-kinase, Smad, transforming growth factor β type I (II) receptor

## Abstract

LeY (Lewis Y) is a difucosylated oligosaccharide carried by glycoconjugates on the cell surface. Elevation of LeY is frequently observed in epithelial-derived cancers and is correlated to pathological staging and prognosis. To study the role of LeY on cancer cells, a stably LeY-overexpressing cell line, RMG-I-H, was developed previously by transfection of the α1,2-fucosyltransferase gene, a key enzyme that catalyzes the synthesis of LeY, into ovarian carcinoma-derived RMG-I cells. Our studies have shown that LeY is involved in the changes in biological behavior of RMG-I-H cells. However, the mechanism is still largely unknown. In this study, we determined the structural relationship and co-localization between LeY and TβRI/TβRII, respectively, and the potential cellular signaling mechanism was also investigated. We found that both TβRI and TβRII contain the LeY structure, and the level of LeY in TβRI and TβRII in RMG-I-H cells was significantly increased. Overexpression of LeY up-regulates the phosphorylation of ERK, Akt and down-regulates the phosphorylation of Smad2/3. In addition, the phosphorylation intensity was attenuated significantly by LeY monoantibody. These findings suggest that LeY is involved in the changes in biological behavior through TGF-β receptors via Smad, ERK/MAPK and PI3K/Akt signaling pathways. We suggest that LeY may be an important composition of growth factor receptors and could be an attractive candidate for cancer diagnosis and treatment.

## Introduction

Aberrant glycosylation expressed in glycosphingolipids and glycoproteins in tumor cells has been implicated as an essential mechanism in defining stage and fate of tumor progression. In ovarian cancer, this abnormality mainly focuses on the type II sugar chain such as changes in type II H antigen, Lewis Y (LeY) and LeX blood group antigen. LeY is mainly expressed during embryogenesis and limits to epithelium and granulocytes in adults under physiological conditions. However, LeY is expressed in most epithelial cancers and elevated expression of LeY has been found in 70–90% of the human carcinomas of epithelial cell origin, including colon, lung, ovarian and breast cancer and the elevated expression level is closely associated with poor prognosis (1–4).

Previously, we transfected the ovarian cancer cell line RMG-I with α1,2-fucosyltransferase (α1,2-FT) gene to obtain stable transfectants, RMG-I-H, that highly express LeY (5,6). Our studies showed that, compared with cells without transfection, RMG-I-H cells have enhanced malignant behavior, a shorter cell cycle, and increased resistance to 5-fluorouracil (5,7,8). In addition, LeY mAb dramatically inhibits cell proliferation and cell adhesion of RMG-I-H cells *in vitro*, and the size and weight of tumors derived from RMG-I-H cells *in vivo* are reduced significantly by preincubation of RMG-I-H cells with anti-LeY mAb (8,9). All these suggest that LeY is involved in the changes in biological behavior of the RMG-I cells.

TGF-β (transforming growth factor-β) belongs to the TGF-β superfamily of growth factors and exerts a diverse range of biological functions including differentiation, proliferation, angiogenesis and immunosuppression. Wang *et al* (10) reported that α1,6-fucosyltransferase gene (*FUT8*) deficient mice have dysregulation of TGF-β receptor activation and downstream signaling and show emphysema-like changes in the lung. By reintroducing *FUT8*, the TGF-β receptor signaling abnormality was rescued. We found previously that 88 genes were changed in RMG-I-H cells by gene chip technique and the altered genes were involved in protein phosphorylation, cell signaling and transcription. Among the genes with modified expression, TGFBI (GenBank ID: BC000097) was significantly up-regulated (11). By immunohistochemical staining and Western blot analysis, we examined the expression of TGF-β1 in nude mouse xenograft tumors and found an increased expression in RMG-I-H cells (9). Because LeY is present on cell surface and may modify the growth factor receptor (12,13), we therefore hypothesized that LeY may be involved in the regulation of TGF-β mediated cell growth as part of TGF-β receptors (TβRs).

In the present investigation, TβRs was selected to study the effects of α1,2-FT on its expression and LeY content. Furthermore, Smad2/3, Smad7, Akt, ERK1/2 and MEK were analyzed as the signaling molecules involved in TβRs signaling. Mock cells transfected with the vector were used as controls. We report for the first time that as an important part of TβRI and TβRII, LeY antigen regulates Smad, ERK1/2 and PI3K pathways though TβRs to participate in development of ovarian cancer.

## Materials and methods

### Cell lines and reagents

The human ovarian cancer cell line, RMG-I, which was originated from the tissues of human ovarian clear cell carcinoma, was donated by Professor M. Iwamori of Tokyo University of Japan. RMG-I-H cell line was established as previously reported (6,7). The RMG-I-C cells transfected with the vector alone were used as controls.

Recombinant human TGF-β1 was from peprotech. Anti-TGFβ RI, anti-TGFβ RII, anti-ERK1, anti-dually phosphorylated ERK (Thr202/Tyr204), anti-MEK-1/2, anti-p-MEK-1/2, anti-Akt, anti-p-Akt, anti-Smad7 antibody, horseradish peroxidase (HRP)-labeled secondary antibodies and protein A/G Plus-Agarose were from Santa Cruz Biotechnology. Anti-Smad2/3 and anti-p-Smad2/3 antibodies were from Abzoom. TRIzol, PrimeScript™ RT reagent kit, SYBR^®^ Premix Ex Taq™ and GAPDH primer (D3702) from Takara. Mouse anti-human LeY mAb was produced by immunization of female BALB/c weanling mice with LeY purified from a SK-LU-3 lung cancer cell line. Antigen preparation was mixed with complete Freunds adjuvant and mice were injected intraperitoneally with 0.2 ml of this preparation on Day 0 and intravenously on Day 21 (14). Harvested immune spleen cells were fused with myeloma cells 4 days after the second immunization to produced hybridomas as described previously (15). When the cells had reached 50% confluency in the majority of wells showing cell growth, the supernatants were collected and assayed by enzyme-linked immunosorbent assay for reaction with synthetic LeY and additionally with a panel of related A, B, H blood group antigens and Lewis antigens (Isosep AB, Tullinge, Sweden and Dextra Laboratories, Reading, UK) as described previously (16). The specific anti-LeY antibody was purified from culture supernatants using conventional ultrafiltration techniques. Protein concentration was determined spectrophotometrically by absorption at 280 nm. Immunoglobulin isotype was identified as of the IgM class by using a MonoAb-ID immunoassay kit (Invitrogen) and electrophoretic analysis (14). The mouse mAb was suspended in buffer containing 0.01 M phosphate-buffered saline, 0.1% sodium azide and 1% bovine serum albumin.

### Cell culture and treatment

The method for cell culture has been described previously (6). For Western blot assays, subconfluent cell layers were rendered quiescent by serum starvation for 12–24 h. Cells were stimulated subsequently by addition of medium containing or lacking TGF-β1 (5 ng/ml) for the specified time period. For inhibition assay, LeY mAb (10 μg/ml) was added for different times (1, 10 and 30 min) before stimulation with TGF-β1.

### Real-time PCR

Total RNA was extracted using trizol reagent. cDNA was synthesized using Takara PrimeScript™ RT reagent Kit. Primers used for amplification: TβRI (158 bp) forward, AGTGTTCTGGCTCCAAATGGTAGT; reverse, GGCCCATGGGTATTCCAGTAATC. TβRII (75 bp) forward, GCAGGTGGGAACTGCAAGAT; reverse, GAAGGACTCA ACATTCTCCAAATTC. Reaction conditions were 37°C for 15 min, 85°C for 5 sec, 4°C for 5 min. The real-time PCR reaction conditions were denature at 94°C for 20 sec, 45 cycles of 94°C for 20 sec and 60 or 58°C for 20 sec in a 20-μl reaction mixture containing SYBR^®^ Premix Ex Taq^TM^ (2X) 10 μl, forward primer (5 μmol/l) 1 μl, reverse primer (5 μmol/l) 1 μl, cDNA 2 μl, dH_2_O 6 μl. GAPDH was used as the endogenous control. The Light Cycler PCR and detection system (Roche Diagnostics, Mannheim, Germany) was used for real-time PCR amplification and Ct value calculation. Once the amplification was completed, the melting curve was analyzed. The change of target gene expression level was calculated using the 2^−ΔΔCT^ method (17).

### Western blot analysis

Cells were rinsed with PBS and 1% of Triton X-100 lysis buffer (20 mM Tris-HCl, pH 7.4, 10 mM EGTA, 10 mM MgCl_2_, 1 mM benzamidine, 60 mM β-glycerophosphate, 1 mM Na_3_VO_4_, 20 mM NaF, 2 μg/ml aprotinin, 5 μg/ml leupeptin, 0.1 mM phenylmethylsulfonyl fluoride) was added. Then centrifuged, and the supernatants were collected. Protein content was measured using the protein assay BCA kit (Beyotime Biotechnology, China) and equal amounts of protein were loaded on SDS-PAGE gels. Subsequently, proteins were transferred to PVDF membranes (Millipore, Beaford, MA) and were probed with antibodies (1:1000). Immunoreactive bands were visualized by chemiluminescence (ECL; Pierce) using a secondary antibodies (1:8000).

### Membrane protein isolation and immunoprecipitation

Membrane proteins were extracted and concentrated with Mem-PER^®^ eukaryote membrane protein extraction kit and Pierce^®^ SDS-PAGE Sample Prep Kit (Pierce, Rockford, USA). Membrane proteins were then incubated at 4°C for 2 h with TβRI or TβRII antibody. The immune complexes were isolated by stirring the mixture at 4°C overnight with Protein A/G Plus-Agarose. Thereafter, the samples were loaded onto 10% SDS-PAGE for Western blotting with the procedure described above. The LeY antibody (1:2000) was used to detect the expression of LeY in TβRI and TβRII. TβRI and TβRII antibodies (1:1000) were used to detect the expression of TβRI and TβRII, respectively.

### Immunofluorescence-staining procedure

Cells were fixed with 4% paraformaldehyde. After blocking with normal goat serum, cells were incubated with LeY and TβRI (or TβRII) antibodies (1:100) for 1 h at RT. Cells were then incubated with goat anti-mouse tetramethylrhodamine isothiocyanate (TRITC) conjugated antibody and goat anti-rabbit fluorescein isothiocyanate (FITC) labeled antibody (1:200) (Zhongshan Biotech, Beijing, China) for 1 h at RT in dark. 4,6-Diamidino-2-phenylindole (DAPI) was used to stain the nuclei at RT for 1 min. Stained slide was observed with a laser confocal microscope (C1-SI; Nikon, Tokyo, Japan). Data were collected using a computer and the digital images were generated.

### Statistical analysis

The SPSS 12.0 statistical analysis software was used, while the analysis of variance was employed. p<0.05 was regarded as with statistical significance.

## Results

### TβRI expression does not change, while TβRII expression is elevated

**Figure 1:**
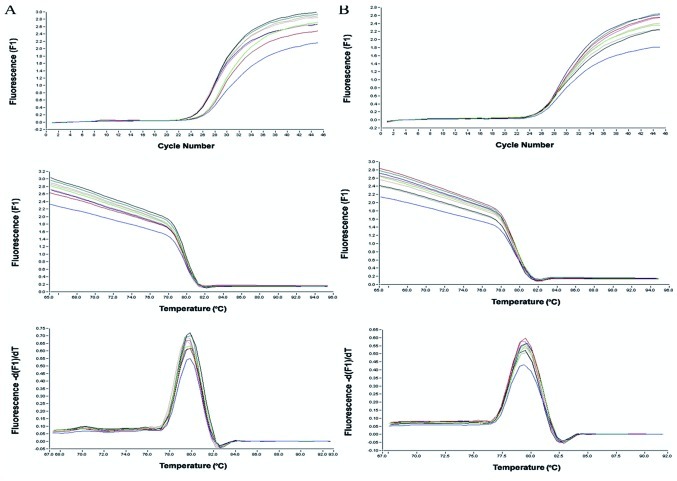
Determination of the expression of TβRI mRNA (A) and TβRII mRNA (B) of cells before and after transfection using real-time PCR.

Cells were subjected to real-time PCR analyses to assess the mRNA levels of TβRI and TβRII. The results show that the TβRII mRNA levels in RMG-I-H cells were 1.69- and 1.74-fold (>1.3-fold) higher than that in the RMG-I and RMG-I-C cells, respectively. However, TβRI mRNA level in RMG-I-H cells was 1.03- and 1.00-fold compared to that in the RMG-I and RMG-I-C cells, respectively (Fig. 1).

### Expression of LeY in TβRI and TβRII on the cell membrane is elevated

**Figure 2:**
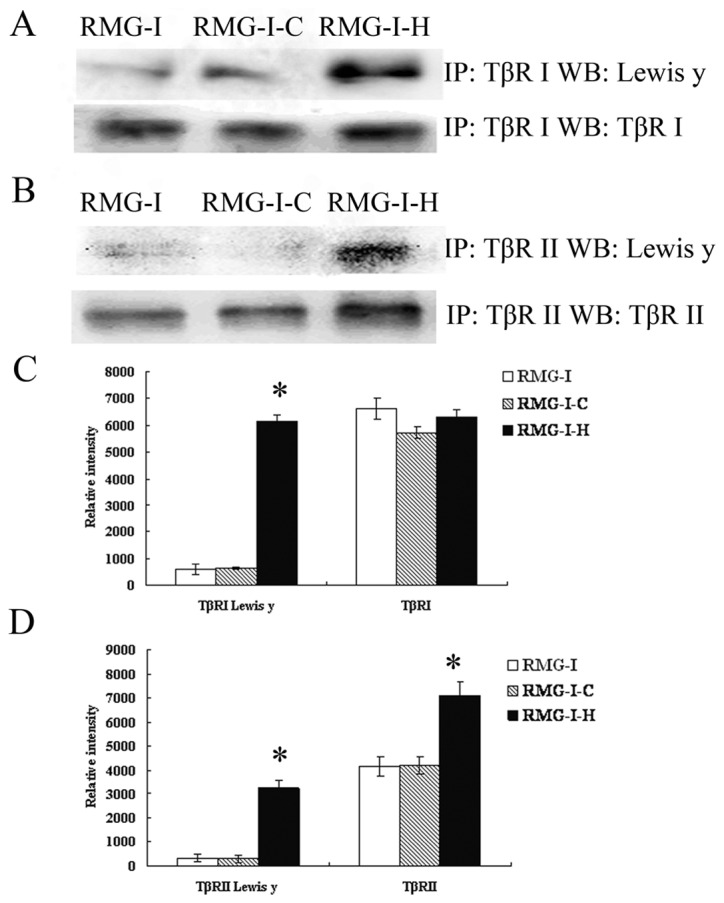
Expression of LeY in TβRI and TβRII. (A, B) Following immunoprecipitation with TβRI and TβRII antibodies, Western blot analysis was performed to analyze the expression of LeY antigen and the level of TβRI, TβRII. (C, D) The relative intensity of protein levels are expressed as means in bar graphs, significant differences from RMG-I and RMG-I-C cells are noted as ^*^p<0.01.

To estimate the expression of the LeY oligosaccharide in TGF-β receptors, we performed a series of immunoprecipitation experiments to determine whether LeY mAb would bind to membrane extracts precipitated by TβRI and TβRII antibody. After SDS-PAGE, followed by immunoblotting, anti-LeY mAb stained the 53/70 kDa protein bands precipitated by TβRI and TβRII antibody, respectively. The results showed that both TβRI and TβRII contained LeY structures, and TβRI and TβRII showed absolute or relative increase in the content of LeY in the RMG-I-H cells, respectively (Fig. 2).

### Co-localization of TGF-β receptors and LeY on RMG-I-H cell surface

**Figure 3:**
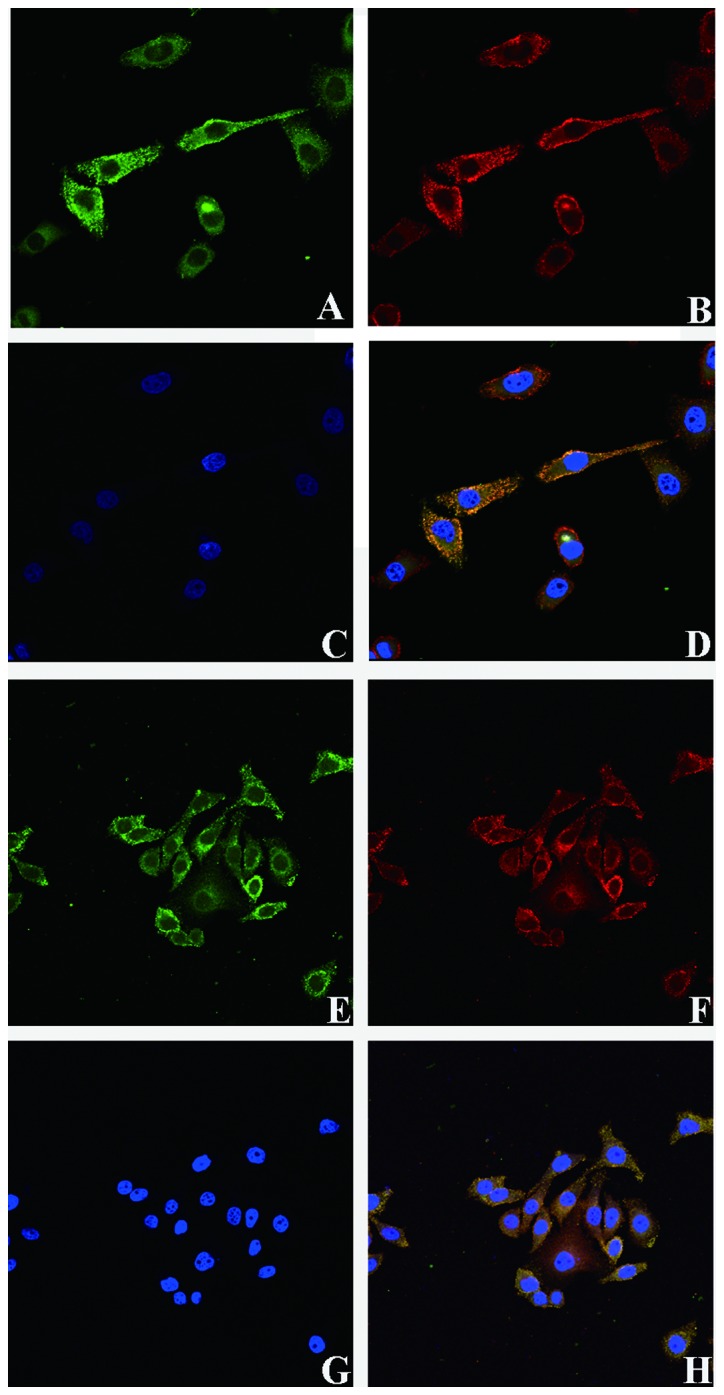
Confocal microscopy analysis of TGF-β receptor (TβRI and TβRII) and LeY co-localization in RMG-I-H cells. Cells were fixed and stained for LeY (red) and TβRI (or TβRII) (green). (A, E) Localization of TβRI and TβRII in RMG-I-H cells, respectively. (B, F) Localization of Lewis Y. (D, H) Co-localization (yellow) of LeY and TβRI (or TβRII) is indicated by white arrows in the merged images. (C, G) Nuclear stained by DAPI (blue).

Immunoprecipitation assay showed that TβRI and TβRII contain LeY structure, we verified the spatial orientation of TGF-β receptors and LeY by immunofluorescence double staining. TβRI and TβRII were labeled with FITC and LeY antigen was labeled with TRITC. Images were scanned using a confocal microscope in serial Z-sections and then overlaid. The results clearly showed that TβRI and TβRII in green fluorescence were mainly localized on the cell membrane with a small amount localized in the cytoplasm (Fig. 3A and E); LeY antigen in red fluorescence was mainly on the cell membrane (Fig. 3B and F). As shown in the merged figures, spatial co-localization of TGF-β receptors and LeY exhibited yellow fluorescence (Fig. 3D and H, white arrow).

### LeY down-regulates TGF-β/Smad pathways

**Figure 4:**
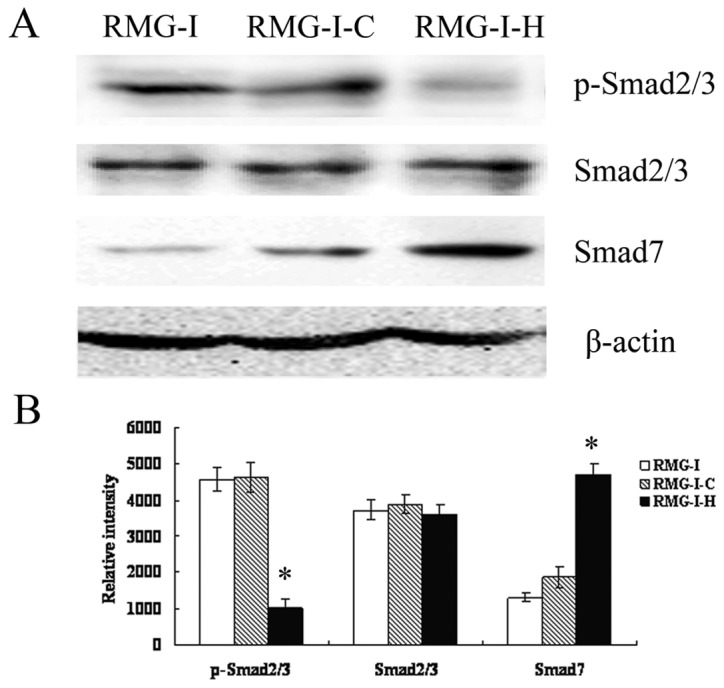
Changes in the Smad pathway. (A) Western blot analysis was performed to analyze the expression of p-Smad2/3, Smad2/3 and Smad7 proteins in RMG-I, RMG-I-C and RMG-I-H cells. (B) Relative intensity of p-Smad2/3, Smad2/3 and Smad7 protein levels were expressed as means in bar graphs. Significant differences from RMG-I and RMG-I-C cells were noted as ^*^p<0.01.

To further characterize the effect of LeY on TGF-β/Smad pathway, Western blot analysis was performed to analyze the expression of Smad2/3, p-Smad2/3 and Smad7 (inhibitory Smad), which are key members of TGF-β1/Smad, in cells before and after α1,2-FT gene transfection after TGF-β1 stimulation (5 ng/ml). The results showed that total protein levels of Smad2/3 did not change significantly (p>0.05), however, p-Smad2/3 level was significantly decreased in RMG-I-H cells compared to RMG-I and RMG-I-C cells. The expression of Smad7 was up-regulated in RMG-I-H cells (Fig. 4). The results indicated that LeY down-regulated the activation of TGF-β1/Smad pathway.

### LeY up-regulates TGF-β1-dependent ERK and PI3K pathways

**Figure 5:**
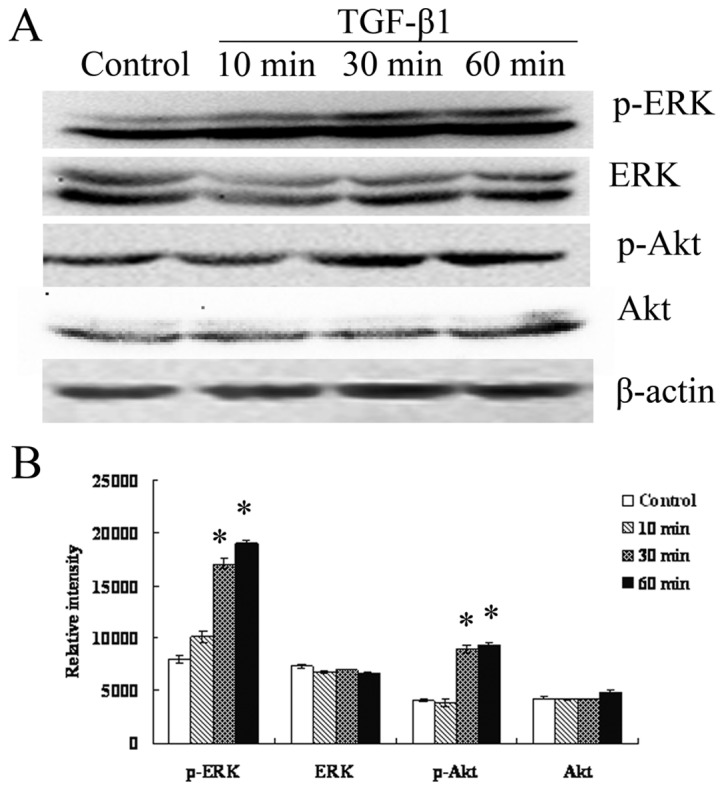
TGF-β1 activates ERK/MAPK and PI3K pathways in RMG-I-H cells. (A) Western blot analysis was performed to detect the expression of p-ERK, ERK, p-Akt and Akt in RMG-I-H cells at different time-points after TGF-β1 stimulation. (B) Relative intensity of p-ERK, ERK, p-Akt and Akt protein were expressed as means in bar graphs, significant differences from control cells were noted as ^*^p<0.01.

**Figure 6:**
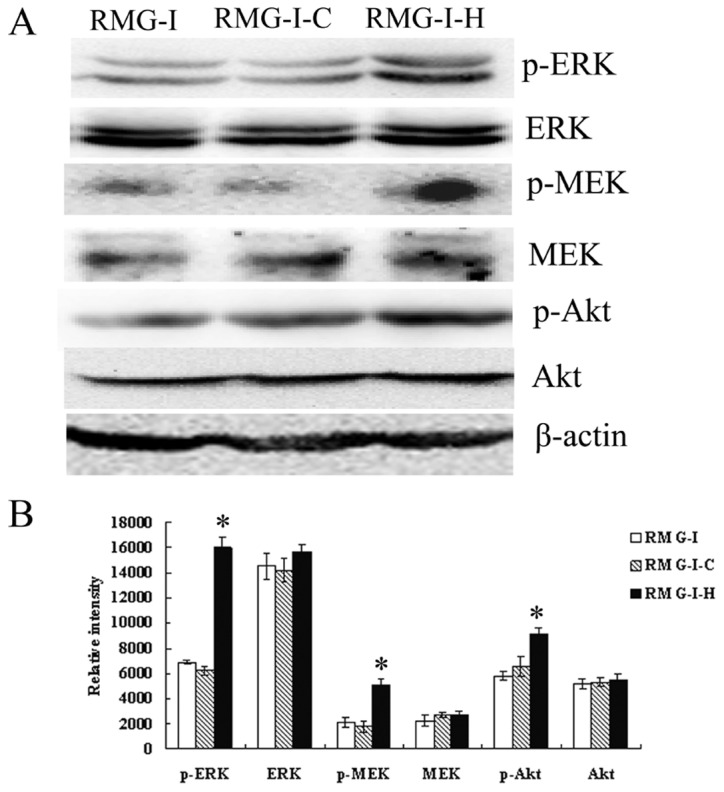
Changes in expression levels of elements of ERK and PI3K signaling pathways. (A) Changes of the expression and phosphorylation levels of MEK, ERK, Smad2/3 and Akt in RMG-I, RMG-I-C and RMG-I-H cells. (B) Relative intensity of elements of ERK and PI3K signaling pathways protein levels were expressed as means in bar graphs. Significant differences from RMG-I and RMG-I-C cells were noted as ^*^p<0.01 (p-ERK and p-MEK) and ^*^p<0.05 (p-Akt).

Given that ERK/MAPK and PI3K are two important pathways that can also be activated by TGF-β in some cell types (18–21), we first analyzed the expression of ERK1/2 and Akt in TGF-β1 stimulated RMG-I-H cells following serum starvation by Western blot analysis to determine whether TGF-β can activate ERK/MAPK and PI3K in RMG-I-H cells. As shown in Fig. 5, expression of EKR1/2 and Akt did not change (p>0.05), however, p-EKR1/2, p-Akt increased over time, suggesting TGF-β1 indeed activate the ERK and PI3K pathways in RMG-I-H cells. Then we compared the expression of ERK1/2, MEK1/2 and Akt in cells before and after α1,2-FT gene transfection, the results showed that the total protein levels of ERK1/2, MEK1/2 and Akt did not change significantly among cells (p>0.05), but the p-ERK1/2, p-MEK1/2 and p-Akt levels were significantly increased in RMG-I-H cells than those in RMG-I and RMG-I-C cells (Fig. 6). These results demonstrate that LeY up-regulated the activation of TGF-β1 mediated ERK and PI3K pathways.

### LeY mAb inhibits TGF-β1-dependent activation of Smad, ERK and PI3K pathways

**Figure 7:**
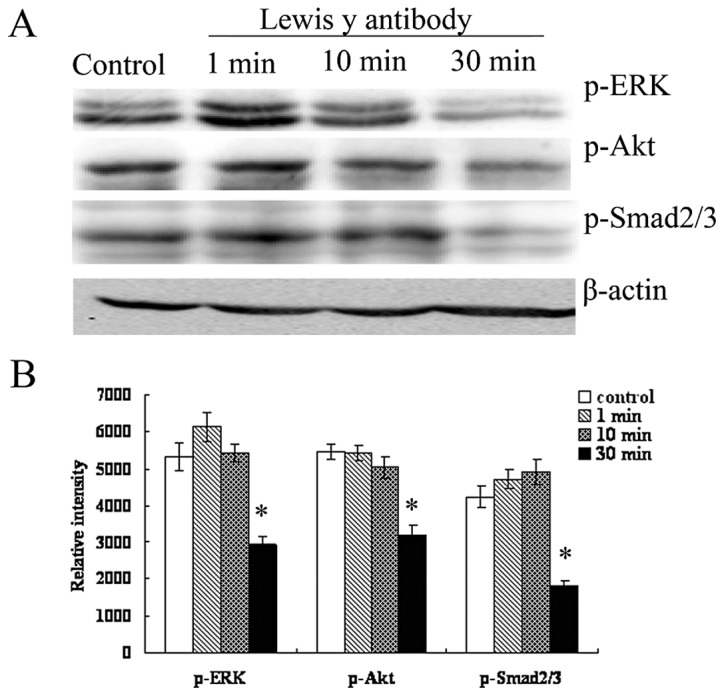
The effect of LeY antibody on the expression of p-ERK, p-Smad2/3 and p-Akt. (A) A Western blot analysis was performed to measure changes in the phosphorylation levels of ERK, Smad2/3 and Akt at different time-points after antibody blocking. (B) Relative intensity of p-ERK, p-Smad2/3 and p-Akt protein levels were expressed as means in bar graphs. Significant differences from control cells were noted as ^*^p<0.01.

The expression of phosphorylated ERK1/2, Smad2/3 and Akt in RMG-I-H cells at different time-points (1, 10 and 30 min) after treatment with LeY mAb (10 μg/ml) was analyzed by Western blotting. The results show that the expression levels of p-Smad2/3, p-ERK1/2 and p-Akt decreased over the time of antibody treatment (Fig. 7), indicating LeY antigen involvement in the TGF-β1-dependent Smad, ERK and PI3K pathways.

## Discussion

TGF-β is a member of the growth factor superfamily that has multiple biological functions. During the early stage of tumorigenesis, TGF-β1 functions as an important tumor suppressor to inhibit tumor cell proliferation. However, after the cells become resistant to TGF-β induced inhibition, TGF-β promotes tumor development (22–24). It has been reported that all epithelial tumors (>85% of human cancers) can become resistant to TGF-β mediated growth inhibition (23,25,26) including ovarian cancer (27,28). In gastric cancer, colon cancer and pancreatic cancer, loss of the sensitivity to TGF-β inhibition has mainly been attributed to mutations in TβRII (29,30) and in downstream molecules such as Smad2 and Smad4 (31–33). However, in ovarian cancers, mutations in the TGF-β receptor and Smad are rare (34–36). Therefore, it remains likely that there are other mechanisms underlying the interference of the TGF-β signaling pathway.

LeY antigen is carried by glycoconjugates on cell surface, this result in the modification of cell surface receptors by the LeY. We examined the structural relationship of LeY and TβRs and found that both TβRI and TβRII contain LeY structures (Figs. 2 and 3). Meanwhile, we found that the expression of LeY was significantly increased in RMG-I-H cells. Since it is known that the carbohydrate moieties on the cell surface can be changed by altering the expression of glycosyltransferase, which in turn affect the receptor’s functionality (37–39), we examined the effect of LeY on TGF-β/Smad pathway. We found that although the expression of Smad2/3 did not change significantly in LeY overexpressing cells, the level of p-Smad2/3 was down-regulated and Smad7 expression levels were significantly elevated (Fig. 4), suggestting that activation of TGF-β/Smad pathway was inhibited in LeY overexpressing cells.

We further tested the activation of MAPK and PI3K pathway to judge whether TGF-β/Smad pathway was inhibited by excessive activation of MAPK and PI3K. The results showed that the ERK/MAPK and PI3K pathways were not only activated in RMG-I-H cells, but also up-regulated in RMG-I-H cells compared to RMG-I and RMG-I-C cells. Using LeY mAb to block the function of LeY, we found that phospho-Smad2/3, phospho-ERK1/2 and phospho-Akt levels all decreased over time in RMG-I-H cells (Fig. 7). These results prove that LeY antigen is involved in the regulation of TGF-β mediated activation of Smad, MAPK and PI3K pathway. Therefore, we came to the conclusion that LeY is involved in regulating Smad, MAPK and PI3K signaling pathways as a key structure on TGF-β receptor. The ways LeY possibly regulates signal transduction are: i) LeY alters the amount of TGF-β that binds to TGF-β receptors and/or the affinity between the TGF-β receptors and TGF-β, leading to the change of activation level of TGF-β signaling; or ii) LeY changes the 3-D conformation of TβRI and TβRII, which results in a relatively high number of TGF-β receptors on cell surface by weakening the receptor endocytosis in cells, and also increases the sensitivity of cells to TGF-β stimulation; iii) i) and/or ii) lead to excessive activation of MAPK and PI3K, which inhibited the activation of TGF-β/Smad pathway.

The interaction of Smad and non-Smad signaling determines the ultimate response of cells to TGF-β. The mechanism underlying the interaction and regulation between the ERK (and/or PI3K) pathways and the Smad pathway warrants further study. Due to the important negative regulatory function of Smad7 in Smad pathway, its role in regulation of MAPK and PI3K pathways has gained much attention. Dowdy *et al* (40) found that TGF-β-activated protein kinase TAK1 activates ER81 via the p-38MAPK pathway and modulates Smad7 transcription. Ohashi *et al* reported that TGF-β activates the Smurf2 (regulates the Smad pathway by degrading Smad2 and Smad7) promoter through Smad-independent PI3K/Akt pathway and up-regulates Smurf2 expression (41). However, in PC-3U prostate cancer cells, Smad7, by promoting the interaction between receptor and MKK3 and p38, is involved in the activation of p38 by TGF-β1 (42,43). Mazars *et al* also verified that both transient and stable transfected Smad7 can induce strong and durable activation of JNK, and speculated that Smad7 activates JNK by direct interaction with JNK upstream molecules (44).

In conclusion, this study is the first to demonstrate that LeY antigen, as an important component in TβRI and TβRII, participates the development of ovarian cancer by regulating TGF-β1-dependent Smad, ERK and PI3K pathways. It provides the rational support for targeted treatment of LeY, and opens a new avenue for exploring the mechanism underlying the resistance of cancer cells to TGF-β induced cell inhibition. Moreover, an interesting suggestion raised by the studies is that LeY antigen may exist in most growth factor receptors in many kinds of cancers and affect cell development via receptor signaling, which will make LeY an attractive candidate for cancer diagnosis and treatment.

## References

[1] Baldus SE, Mönig SP, Zirbes TK (2006). Lewis(y) antigen (CD174) and apoptosis in gastric and colorectal carcinomas: correlations with clinical and prognostic parameters. Histol Histopathol.

[2] Kuemmel A, Single K, Bittinger F (2007). The prognostic impact of blood group-related antigen Lewis Y and the ABH blood groups in resected non-small cell lung cancer. Tumour Biol.

[3] Chhieng DC, Rodriguez-Burford C, Talley LI (2003). Expression of CEA, Tag-72, and Lewis-Y antigen in primary and metastatic lesions of ovarian carcinoma. Hum Pathol.

[4] Madjd Z, Parsons T, Watson NF, Spendlove I, Ellis I, Durrant LG (2005). High expression of Lewis y/b antigens is associated with decreased survival in lymph node negative breast carcinomas. Breast Cancer Res.

[5] Iwamori M, Tanaka K, Kubushiro K (2005). Alterations in the glycolipid composition and cellular properties of ovarian carcinoma-derived RMG-1 cells on transfection of the α1,2-fucosyltransferase gene. Cancer Sci.

[6] Lin B, Hao YY, Wang DD, Zhu LC, Zhang SL, Saito M, Iwamori M (2008). Transfection of α1,2-fucosyltransferase gene increases the antigenic expression of Lewis y in ovarian cancer cell line RMG-I. Acta Acad Med Sin.

[7] Hao YY, Lin B, Zhao Y (2008). Alpha1,2-fucosyltransferase gene transfection influences on biological behavior of ovarian carcinoma-derived RMG-I cells. Fen Zi Xi Bao Sheng Wu Xue Bao.

[8] Liu J, Lin B, Hao Y (2009). Lewis y antigen promotes the proliferation of ovarian carcinoma-derived RMG-I cells through the PI3K/Akt signaling pathway. J Exp Clin Cancer Res.

[9] Li F, Lin B, Hao Y (2010). Lewis y promotes growth and adhesion of ovarian carcinoma-derived RMG-I cells by upregulating growth factors. Int J Mol Sci.

[10] Wang X, Inoue S, Gu J (2005). Dysregulation of TGF-β1 receptor activation leads to abnormal lung development and emphysema-like phenotype in core fucose-deficient mice. Proc Natl Acad Sci USA.

[11] Zhu LC, Lin B, Hao YY, Li FF, Diao B, Zhang SL (2008). Impact of α1,2-fucosyltransferase gene transfection on cancer-related gene expression profile of human ovarian cancer cell line RMG-1. Ai Zheng.

[12] Basu A, Murthy U, Rodeck U, Herlyn M, Mattes L, Das M (1987). Presence of tumor-associated antigens in epidermal growth factor receptors from different human carcinomas. Cancer Res.

[13] Klinger M, Farhan H, Just H (2004). Antibodies directed against Lewis-Y antigen inhibit signaling of Lewis-Y modified ErbB receptors. Cancer Res.

[14] Kosunen TU, Bång BE, Hurme M (1984). Analysis of *Campylobacter jejuni* antigens with monoclonal antibodies. J Clin Microbiol.

[15] Bång BE, Hurme M, Juntunen K, Mäkelä O (1981). Studies of monoclonal and polyclonal anti-digoxin antibodies for serum digoxin radioimmunoassay. Scand J Clin Lab Invest.

[16] Moran AP, Knirel YA, Senchenkova SN, Widmalm G, Hynes SO, Jansson PE (2002). Phenotypic variation in molecular mimicry between *Helicobacter pylori* lipopolysaccharides and human gastric epithelial cell surface glycoforms. Acid-induced phase variation in Lewisx and Lewisy expression by *H pylori* lipopolysaccharides. J Biol Chem.

[17] Livak KJ, Schmittgen TD (2001). Analysis of relative gene expression data using real-time quantitative PCR and the 2^−ΔΔCT^ method. Methods.

[18] Yu L, Hébert MC, Zhang YE (2002). TGF-β receptor-activated p38 MAP kinase mediates Smad-independent TGF-β responses. EMBO J.

[19] Derynck R, Zhang YE (2003). Smad-dependent and Smad-independent pathways in TGF-β family signaling. Nature.

[20] Wilkes MC, Mitchell H, Penheiter SG (2005). Transforming growth factor-β activation of phosphatidylinositol 3-kinase is independent of Smad2 and Smad3 and regulates fibroblast responses via p21-activated kinase-2. Cancer Res.

[21] Zhang YE (2009). Non-Smad pathways in TGF-β signaling. Cell Res.

[22] Pasche B (2001). Role of transforming growth factor beta in cancer. J Cell Physiol.

[23] Elliott RL, Blobe GC (2005). Role of transforming growth factor beta in human cancer. J Clin Oncol.

[24] Tian M, Schiemann WP (2009). The TGF-β paradox in human cancer: an update. Future Oncol.

[25] Fynan TM, Reiss M (1993). Resistance to inhibition of cell growth by transforming growth factor-beta and its role in oncogenesis. Crit Rev Oncol.

[26] Massagué J, Blain SW, Lo RS (2000). TGFβ signaling in growth control, cancer, and heritable disorders. Cell.

[27] Yamada SD, Baldwin RL, Karlan BY (1999). Ovarian carcinoma cell cultures are resistant to TGF-β1-mediated growth inhibition despite expression of functional receptors. Gynecol Oncol.

[28] Hu W, Wu W, Nash MA, Freedman RS, Kavanagh JJ, Verschraegen CF (2000). Anomalies of the TGF-beta postreceptor signaling pathway in ovarian cancer cell lines. Anticancer Res.

[29] Chang J, Park K, Bang YJ, Kim WS, Kim D, Kim SJ (1997). Expression of transforming growth factor β type II receptor reduces tumorigenicity in human gastric cancer cells. Cancer Res.

[30] Grady WM, Rajput A, Myeroff L, Liu DF, Kwon K, Willis J, Markowitz S (1998). Mutation of the type II transforming growth factor-β receptor is coincident with the transformation of human colon adenomas to malignant carcinomas. Cancer Res.

[31] Hahn SA, Schutte M, Hoque AT (1996). DPC4, a candidate tumor suppressor gene at human chromosome 18q21.1. Science.

[32] Miyaki M, Iijima T, Konishi M (1999). Higher frequency of Smad4 gene mutation in human colorectal cancer with distant metastasis. Oncogene.

[33] Eppert K, Scherer SW, Ozcelik H (1996). MADR2 maps to 18q21 and encodes a TGFβ-regulated MAD-related protein that is functionally mutated in colorectal carcinoma. Cell.

[34] Wang D, Kanuma T, Takama F (1999). Mutation analysis of the smad3 gene in human ovarian cancers. Int J Oncol.

[35] Vincent F, Nagashima M, Takenoshita S, Khan MA, Gemma A, Hagiwara K, Bennett WP (1997). Mutation analysis of the transforming growth factor-β type II receptor in human cell lines resistant to growth inhibition by transforming growth factor-β. Oncogene.

[36] Wang D, Kanuma T, Mizumuma H, Ibuki Y, Takenoshita S (2000). Mutation analysis of the Smad6 and Smad7 gene in human ovarian cancers. Int J Oncol.

[37] Dettke M, Pálfi G, Loibner H (2000). Activation-dependent expression of the blood group-related lewis Y antigen on peripheral blood granulocytes. J Leukoc Biol.

[38] Farhan H, Schuster C, Klinger M (2006). Inhibition of xenograft tumor growth and down-regulation of ErbB receptors by an antibody directed against Lewis Y antigen. J Pharmacol Exp Ther.

[39] Wang QY, Zhang Y, Chen HJ, Shen ZH, Chen HL (2007). Alpha 1,3-fucosyltransferase-VII regulates the signaling molecules of the insulin receptor pathway. FEBS J.

[40] Dowdy SC, Mariani A, Janknecht R (2003). HER2/Neu- and TAK1-mediated up-regulation of the transforming growth factor β inhibitor Smad7 via the ETS protein ER81. J Biol Chem.

[41] Ohashi N, Yamamoto T, Uchida C (2005). Transcriptional induction of Smurf2 ubiquitin ligase by TGF-β. FEBS Lett.

[42] Edlund S, Bu S, Schuster N (2003). Transforming growth factor-β1 (TGF-β)-induced apoptosis of prostate cancer cells involves Smad7-dependent activation of p38 by TGF-β-activated kinase 1 and mitogen-activated protein kinase kinase 3. Mol Biol Cell.

[43] Iwai T, Murai J, Yoshikawa H, Tsumaki N (2008). Smad7 inhibits chondrocyte differentiation at multiple steps during endochondral bone formation and down-regulates p38 MAPK pathways. J Biol Chem.

[44] Mazars A, Lallemand F, Prunier C (2001). Evidence for a role of the JNK cascade in Smad7-mediated apoptosis. J Biol Chem.

